# Bridging the gap between *in vitro* and *in vivo*: Dose and schedule predictions for the ATR inhibitor AZD6738

**DOI:** 10.1038/srep13545

**Published:** 2015-08-27

**Authors:** Stephen Checkley, Linda MacCallum, James Yates, Paul Jasper, Haobin Luo, John Tolsma, Claus Bendtsen

**Affiliations:** 1 AstraZeneca, Alderley Park, Macclesfield, SK10 4TG. UK; 2 RES Group Inc. Boston, MA. USA

## Abstract

Understanding the therapeutic effect of drug dose and scheduling is critical to inform the design and implementation of clinical trials. The increasing complexity of both mono, and particularly combination therapies presents a substantial challenge in the clinical stages of drug development for oncology. Using a systems pharmacology approach, we have extended an existing PK-PD model of tumor growth with a mechanistic model of the cell cycle, enabling simulation of mono and combination treatment with the ATR inhibitor AZD6738 and ionizing radiation. Using AZD6738, we have developed multi-parametric cell based assays measuring DNA damage and cell cycle transition, providing quantitative data suitable for model calibration. Our *in vitro* calibrated cell cycle model is predictive of tumor growth observed in *in vivo* mouse xenograft studies. The model is being used for phase I clinical trial designs for AZD6738, with the aim of improving patient care through quantitative dose and scheduling prediction.

In the pharmaceutical industry, pharmacokinetic and pharmacodynamic (PK-PD) modeling is used to predict dose and scheduling for pre-clinical and clinical studies^1^,^2^,^3^, employing compartmental models and empirical dose to efficacy relationships^4^,^5^,^6^. However, complex diseases such as cancer involves understanding of both biomarker kinetics and tumor regression to understand mode of action as well as drug efficacy, and this combination of biomarker kinetics and tumor growth is often not incorporated into standard PK-PD modeling approaches^5^,^7^,^8^.

Mechanism-based PK-PD modeling has been shown to be informative when investigating non-linear dynamical systems^1^,^4^,^9^,^10^,^11^, and cell cycle models have been used to investigate macroscopic population dynamics when the microscopic underlying interactions cannot be fully characterized^12^,^13^,^14^,^15^. In this study, an existing PK-PD model of tumor growth published by Evans *et al.* (2013) was extended with mechanistic modeling, supported by high content biology to develop an *in vitro* calibrated model for predicting tumor growth and biomarker kinetics in response to treatment with AZD6738 which targets the DNA damage response (DDR) pathway^16^,^17^.

The DDR pathway enables eukaryotic cells to repair DNA damage occurring in response to a variety of endogenous and exogenous sources^18^. Cells are capable of responding to both single and double stranded DNA breaks using two inter-connected signaling pathways involving ATR and ATM, respectively^19^,^20^,^21^. Single stranded breaks occur primarily during DNA synthesis in S phase, resulting in aberrant replication forks containing single stranded DNA that accumulate and induce the DDR (replication stress), with ATR facilitating repair^19^,^22^,^23^,^24^. Double stranded breaks can occur in S phase through collapsed replication forks, or throughout the cell cycle due to external sources of damage such as ionizing radiation (IR)^23^,^25^. Cross-talk has been observed between components of the ATM and ATR pathways and specificity of each for the repair of double and single stranded breaks has yet to be fully elucidated^26^,^27^.

Many diseases, including ataxia telangiectasia (AT), chronic lymphocytic leukemia (CLL), and colorectal cancer (CRC) are associated with mutations in ATM^28^,^29^, leaving ATR as the primary mediator of repair. Inhibition of DNA damage detection can lead to cell cycle arrest or mitotic spindle catastrophe^19^,^30^. The former occurs upon stalling of the replication fork, the latter if checkpoints are dysfunctional because of, e.g. mutations^31^.

AZD6738 is a selective inhibitor of ATR^16^,^32^ and it is hypothesized that application as therapy in an ATM deficient setting will result in deficiency in both DDR pathways, inhibiting DNA damage signaling resulting in cell death if damage is sufficiently high, and abrogation of the G2/M checkpoint with consequent genomic instability during mitosis (mitotic spindle catastrophe)^16^,^18^,^19^.

The LoVo cell line is an ATM deficient model cell line, derived from a metastatic adenocarcinoma of the colon^33^, used both *in vitro* and *in vivo* (LoVo derived xenograft) to investigate the physiological response to dosing with AZD6738^16^,^32^. The LoVo cell line is ATM pathway deficient due to mutation in MRE11, preventing formation of the MRN complex and subsequent recruitment of ATM to sites of DNA damage to initiate repair^34^.

Ser-139 phosphorylated histone 2AX (*γ*H2AX), a well documented biomarker of DNA damage^35^,^36^, was used to measure DNA damage incurred by the cell population as a result of exposure to therapy, and correlated with effect on tumor growth measured by total cell count *in vitro*. In addition to monotherapy, AZD6738 is considered in combination with IR, sometimes used as standard of care to induce DNA damage and promote cell death.

Measuring the many biochemical interactions involved in the DDR is beyond the scope of this study. Large cell cycle models such as those published by John Tyson and Bela Novák^37^,^38^ are difficult to parameterize for specific oncology cell lines, and existing models of tumor growth such as those published by Frieboes (2013) and Lee (2013) do not contain microscopic details that could be used to link DDR biomarker kinetics with cell fate^39^,^40^. Also, detailed studies of single cell dynamics such as the work published by, e.g. the White and Lahav groups, have made substantial contribution to understanding the dynamical behaviors of the interactions within biochemical networks, however it is difficult to translate these complex dynamics into a response in a multi-cellular *in vivo* tumor model, in order to link mode of action and drug efficacy in a cell population for clinical trial design^41^,^42^.

Therefore we decided to extend an existing macroscopic tumor growth model published by Evans (2014) with microscopic detail of the cell cycle, such as those published by Altinok *et al.* (2011) and Hamed *et al.* (2013)^12^,^14^. The extended Evans model provides a multi-scale model of the xenograft, incorporating tumor growth with mechanistic representation of the pharmacodynamic effect of AZD6738 on the cell population under monotherapy and in combination with IR. We here show how this model is useful in informing the initial clinical studies for AZD6738.

## Results

### A mechanistic model of the cell cycle incorporating AZD6738

**Figure 1:**
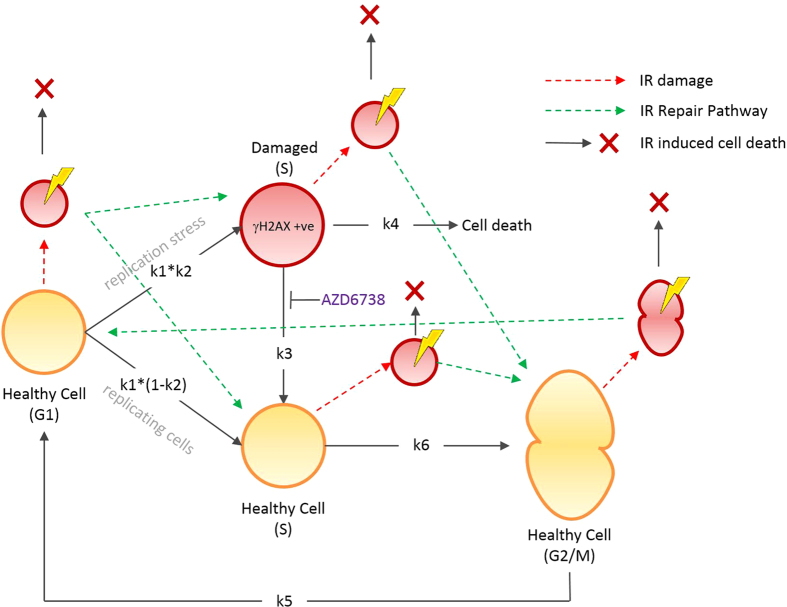
Diagrammatic representation of the cell cycle model incorporating replication stress, IR damage and repair. Replication stress occurs by transition to the S damaged (“*γ*H2AX +ve”) population at rate k1*k2 (or continue through the cell cycle at rate k1*(1-*k2*), and repair is inhibited through reaction *k3* in the presence of AZD6738 resulting in cell death at rate *k4*. IR damage in simulated by instantaneous transition to IR damaged compartments of all cycle phases (red compartments with yellow flashes), resulting in repair or cell death).

A mathematical model of the cell cycle, incorporating DNA damage and repair was formulated as a set of ordinary differential equations (see supplementary material). The model represents the transition of a population of cells through the G1, S and G2/M phases with additional DNA damaged states for replication stress and IR induced damage for the relevant cell cycle phase (Fig. 1). Replication stress is modeled as an alternative S phase (“S damaged”) with transition back into the cell cycle simulating repair, a reaction which is inhibited by the addition of drug, simulating the mode of action of AZD6738. While replication stress induced DNA damage occurs during S phase and one could model the more detailed dynamics of this phase (including varying degrees of damage and checkpoint monitoring), the approach taken here was to abstract from this level of detail and instead partition the S phase into two populations: damaged or undamaged with transition into either directly from G1.

IR damage occurs throughout the cell cycle and was modeled using alternative G1, S, and G2/M IR damaged phases which also transition back into the corresponding cell cycle phase simulating ATM mediated repair (Fig. 1). Using this approach, ATR mediated repair of replication stress could be modeled as a separate process to ATM mediated repair. For the purposes of this study, cross-talk between repair pathways was not explicitly represented in the model and AZD6738 inhibits only ATR mediated repair reactions^16^.

### The model explains *in vitro* biomarker and cell population kinetics

In order to calibrate the model a number of high content *in vitro* assays were developed. These sought to capture the dynamics of *γ*H2AX (as a biomarker of DNA damage) and cell growth in response to both AZD6738 and IR (see Methods). Parameter estimation was performed using both *in vitro* dose response time course and drug wash-out data sets (see Methods). The model parameters are detailed in Table 2 in the supplementary information (see Supplementary Tables 1 and 2). Parameter identifiability results are detailed in the supplementary information and the analysis indicates the model is largely identifiable, except for parameters *ki* and *k3*. These parameters were found to be highly correlated, however the model predictions were found to be insensitive to the different solutions obtained for these two parameters (see Supplementary Figure 1).

**Figure 2:**
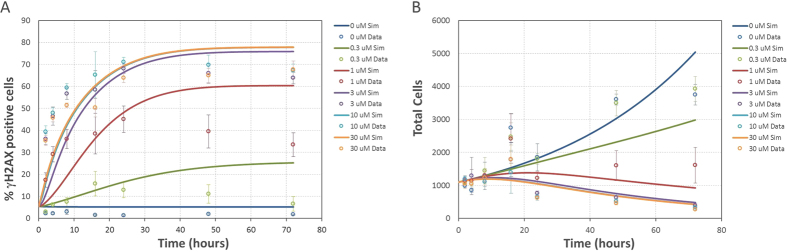
AZD6738 dose response simulations (lines) and experimental data (points). Error bars represent standard deviation of 6 replicates. (**A**) *γ*H2AX experimental measurements and simulation results (R^2^ = 0.77). (**B**) cell count experimental measurements and simulation results (R^2^ = 0.72).

**Figure 3:**
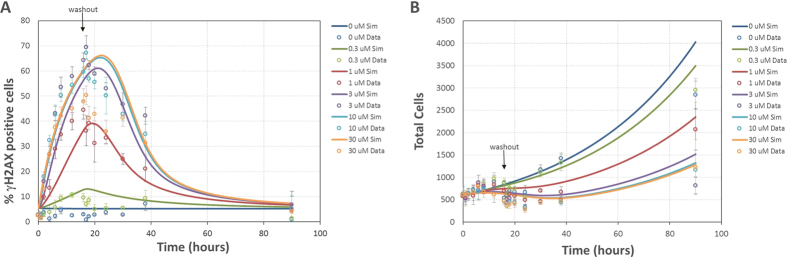
AZD6738 dose response simulations (lines) and experimental data (points). Error bars represent standard deviation of 6 replicates (**A**) *γ*H2AX experimental measurements and simulation results with drug wash-out at 16 hours (R^2^ = 0.87). (**B**) cell count experimental measurements and simulation results with drug wash-out at 16 hours (R^2^ = 0.87).

As shown in Fig. 2, the model is capable of reproducing the *in vitro* AZD6738 dose response behavior of *γ*H2AX and corresponding cell growth. The model is capable of replicating the shift from a low signal at 0.3 μM dose to saturation at doses greater than 3 μM. From the *in vitro* experimental data it was observed that the *γ*H2AX signal was sustained for over 70 hours after initial treatment with AZD6738. To investigate the rate of DNA repair, drug wash-out was performed after the *γ*H2AX signal had been observed to reach maximum signal (approximately after 16 hours). The model is also capable of reproducing the behavior of the drug wash-out experiments for both the *γ*H2AX and cell count data (Fig. 3). The rate of onset of replication stress induced DNA damage, the maximum DNA damage signal for the *γ*H2AX biomarker, and the rate of repair/recovery following drug wash-out are all reproduced by the model.

**Figure 4:**
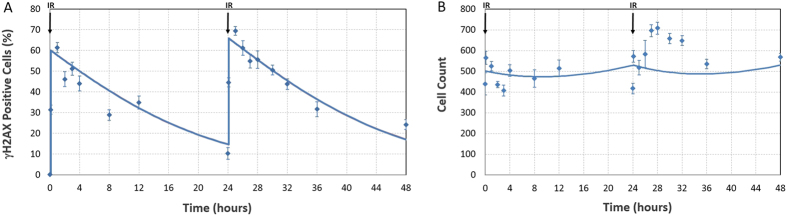
IR response simulations (lines) and experimental data (points). Error bars represent standard deviation of 6 replicates (**A**) *γ*H2AX *in vitro* measurements following 2 Gy IR exposure to LoVo cells. (**B**) cell count *in vitro* measurements following 2 Gy IR exposure to LoVo cells.

Experimental data from IR treated cells were also recapitulated by the model as shown in Fig. 4. After initial DNA damage resulting from exposure to IR, cells transition back into the corresponding undamaged phase and progress through the cell cycle. Using this approach replication stress induced DNA damage and IR induced damage are separated from each other and cells can undergo cell death as a result of entering either the S-damaged or IR damaged cell cycle phases.

To validate the *in vitro* model, image analysis of the cell cycle phase distribution was performed on cells exposed to 10 μM AZD6738 *in vitro* (without wash-out). For treated cells, experimental data shows a directional increase in S phase and decrease in G2/M phase relative to untreated cells. The proportion of cells in each phase in the model corresponds with the experimental observations (see Supplementary Figure 3). The increase in S phase population indicates that cells are, at least in part, attempting to maintain the G2/M checkpoint following treatment with AZD6738 (Supplementary Figures 4, 5 and 6).

In addition, we measured Caspase-3 activation in LoVo cells following 24 hours exposure to AZD6738 and observed a dose dependent response behavior, with corresponding reduction in cell count, indicating that a proportion of the cell population are (in addition to cell cycle arrest) undergoing apoptosis when exposed to drug at concentrations greater than 3 μM (see Supplementary Figures 11 and 12). Jones (2013) *et al.* report cell death occurs by mitotic spindle catastrophe, if sufficient replication stress damage is sustained and our *in vitro* observations provide further evidence that the reduction in the cell population in response to treatment with AZD6738 is a combination of cells in the population exhibiting cell cycle arrest and undergoing apoptosis^16^.

A detailed study of the cell fate decision following exposure to AZD6738 is beyond the scope of this study. Our *in vitro* model is consistent with our observations of the LoVo cell population response since the exact mechanism for cell death is not explicitly modeled, but generally captured as cells delayed in S phase through inhibition of repair, and subsequent exit from the cell population if delayed in the DNA damaged S phase state.

### Translating from *in vitro* to *in vivo* for clinical dose prediction

*In vitro* cell culture is a significantly different environment from that of a solid tumor. To translate the *in vitro* calibrated model into a clinically relevant setting the model was combined with a model of xenograft tumor growth, describing transfer of cells between an actively growing outer shell and inert inner core (see supplementary material)^17^.

**Figure 5:**
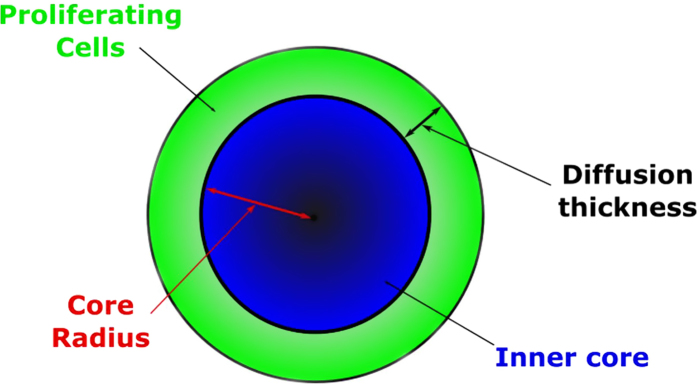
Diagrammatic representation of the inner inert and outer proliferating shell structure of the Evans (2014) model used to represent the structure of a tumor. The proliferating outer shell consists of the cycling cell cycle model and the inner core represents the cell cycle model where progression through the cell cycle is halted. Inhibition of repair by addition of AZD6738 promotes cell death, thereby depleting the outer proliferating shell which is replaced by cells from the inert inner core, reducing the total tumor volume.

To incorporate the cell cycle model into the Evans (2014) model, a copy of the cell cycle model was assigned to the proliferating shell and the non-proliferating core (subscripts s and c in the supplementary material). The cell cycle model for the core was modified so that there was no transition from G1 to S phase to reflect zero proliferation. Cells are assumed to transit to the same phase as they move between the shell and core, with the assumption that cells would complete the cell cycle in the core before remaining in G1. Cells then either progress through the cell cycle, accumulating and repairing DNA damage as described in Fig. 1 if they occupy the outer proliferating shell, or stop their progression through the cell cycle if located in the inert inner core (Fig. 5). Cell death results in depletion of the outer proliferating shell which is replaced with cells from the inert inner core, reducing the overall tumor volume.

The rate of transition between the inner core and outer proliferating shell, the sizes of these compartments, and the initial tumor volume are set as detailed in the original Evans (2014) publication (see Methods, and supplementary materials for the *in vivo* model equations). This approach therefore incorporates a structural element into the *in vitro* calibrated cell cycle model representing tumor growth and enabling translation from the *in vitro* cell assay to *in vivo* xenograft setting.

**Figure 6:**
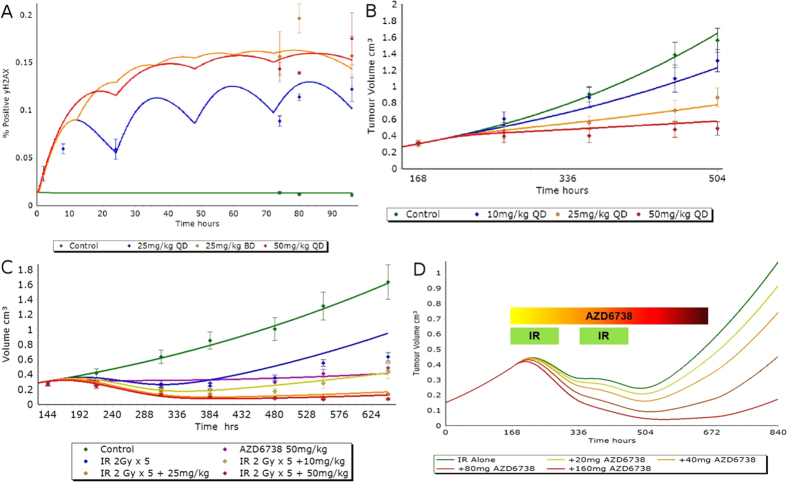
(**A**) Observed (data points, n = 5) and simulated (solid lines) xenograft *γ*H2AX biomarker kinetics following repeat dosing of AZD6738. (**B**) Observed (data points, n = 10) and simulated (solid lines) xenograft tumor growth following repeat dosing of AZD6738. (**C**) Observed (data points, n = 10) and simulated (solid lines) LoVo derived mouse xenograft tumor growth following repeat dosing of AZD6738 and 5 days of 2 Gy ionizing radiation. (**D**) Model simulation of tumor growth under conditions of 5 days of daily 2 Gy radiotherapy for 3 weeks in combination with varying dose of AZD6738 therapy, followed by 1 week of wash-out. Green bars indicate two sets of daily IR exposure over 5 days and the colored bar indicates the 3 weeks of AZD6738 therapy. Error bars represent standard error of the mean. QD = Once daily dosing, BD = Twice daily dosing.

Immunohistochemical studies using *in vivo* mouse xenografts provided measurements of tumor cells with significant *γ*H2AX signal following treatment with AZD6738 (Fig. 6A and Supplementary data file). Setting the initial tumor volume for the *in vivo* model and simulating the drug dosing schedule for 25 mg/kg, the model was able to generate *γ*H2AX profiles that corresponded well with the *in vivo γ*H2AX immunohistochemical data for monotherapy as well as the tumor volume data, without requiring any additional model fitting to incorporate the *in vivo* data sets (Fig. 6A,B). The integrated cell cycle and structural model, calibrated using the *in vitro* data set, was used to simulate tumor growth over time following combination treatment with AZD6738 and IR (Fig. 6C). The model is predictive of xenograft observations without requiring additional calibration to *in vivo* data.

To inform the treatment regime for combining AZD6738 with IR therapy, the predicted human PK for AZD6738, derived using a physiologically based pharmacokinetic (PB-PK) approach was used with the above in silico tumor model to simulate a dosing schedule consisting of two rounds of five days of daily 2 Gy radiotherapy, in combination with three weeks of AZD6738 therapy, followed by 1 week of wash-out (Fig. 6D). From tumor volume simulations an efficacious starting dose of 80 mg/kg AZD6738 was identified, providing clinicians with an indication of the expected rate of tumor regression and recovery (Fig. 6D).

## Discussion

This study demonstrates how mechanistic modeling, supported with *in vitro* experimental data, can be used to extend an *in vivo* mouse xenograft study and PK-PD model of tumor pathophysiology, providing a predictive tool for dosing AZD6738 in the clinic. This approach has gained recent attention in the field of systems pharmacology where mathematical modeling is used to support clinical predictions with *in vivo* studies of drug efficacy^39^,^40^. Our work builds on this approach in the context of DDR drug development, and expands on *in vivo* based studies with high content and higher throughput *in vitro* methods, providing richer data sets for model calibration thus facilitating a model based translation of *in vitro* cell assays to *in vivo* tumor response.

Incorporating biological mechanisms (described at a physiological level corresponding to the *in vitro* methods) enables the extension of empirical PK-PD tumor growth models with representation of the underlying cell population response. In our work this enables model prediction of the tumor cell population, incorporating DNA damage biomarker and cell growth kinetics to predict xenograft tumor response to treatment with AZD6738 as monotherapy and in combination with IR, which was not possible using the original published model^17^.

PK-PD disease progression modeling often relies on low throughput *in vivo* animal model data, with a small sample size and low number of replicates. Existing models of tumor growth focus on changes in tumor size and do not incorporate underlying mechanistic interactions as these are difficult to quantify *in vivo*^6^,^17^,^39^. The approach taken here demonstrates that *in vitro* cell lines can be representative of *in vivo* xenograft models, commonly used in pharmaceutical drug development, providing data higher in both throughput and resolution, ideal for calibrating mechanistic models.

We are able to represent this multi-scale experimental approach of high content *in vitro* data supporting *in vivo* xenograft data with corresponding multi-scale modeling of the microscopic cell population with macroscopic tumor volume dynamics using our combined mechanistic PK-PD model. We are able to mechanistically combine experimental observations of the *γ*H2AX biomarker kinetics with the cell fate decision in the tumor cell population, and by embedding the cell cycle model in place of the PK-PD function in the Evans (2014) model, quantitatively link dose response to AZD6738 to changes in tumor volume.

Further investigation of the components of the DDR pathway is beyond the scope of this study, however it is acknowledged that additional cell cycle and DNA damage biomarkers could be included in subsequent studies to expand our understanding of the mode of action of DDR targeting oncology therapies^43^,^44^. Studies have been published using live cell imaging and demonstrated that detailed information can be obtained for complex dynamics of intracellular signal transduction pathways^42^, however implementing large mechanistic models, such as those published by Tyson and Novák, would add considerable numbers of model reactions that would require significant experimental resource to parameterize in a relevant cell background^45^,^46^. Our model was therefore constructed at a resolution corresponding with the experimental data which could be generated within the time-lines relevant to therapeutic drug discovery, and was appropriate to adding mechanistic detail without digressing from the intended functionality of the tumor model.

The model and experimental assays developed for this work are sufficiently generic to be applied across oncology projects where the drug mode of action can be measured as a perturbation of the cell cycle. The *in vitro* assay described here generated quantitative information on the rate of DNA damage accumulation, maximum *γ*H2AX signal, and rate of repair after wash-out which provides an understanding of the dynamic range of the biomarker signal in response to treatment which can be used in the design of *in vivo* xenograft studies, as well as model calibration. Generating *in vitro* dose response data that can be translated to *in vivo* efficacy via mathematical modeling reduces the dose range required to determine efficacy, reducing the size and duration of animal studies and supporting the pharmaceutical industry’s commitment to the 3Rs (Replacement, Reduction, Refinement) in drug development^47^.

Our model is informing clinicians on the efficacious starting dose for phase I clinical trials of AZD6738 monotherapy and combination with IR. Historically, the treatment regime would be derived empirically, however modeling and simulation provides a quantitative tool for exploring the relationship between dose and scheduling for the required efficacy and tolerability. This is of particular relevance in combination therapy, e.g. with IR for which mucositis is a common side effect, as AZD6738 dosing can be adjusted to avoid toxicity while still maintaining efficacy^48^.

As the field continues to mature and gain acceptance, modeling and simulation can contribute to personalized medicine enabling patients to receive treatment tailored to individual toxicology and tumor regression responses, which will ultimately lead to improved patient care.

## Methods

### *In vitro* experimental methods

#### Cell Culture

LoVo (human colon carcinoma) cells were cultured in DMEM (Sigma) supplemented with 10% fetal bovine serum (Sigma) and maintained at 37 °C, 5% CO_2_ in a humidified atmosphere. Cells were dissociated using Accutase (Sigma) according to the manufacturer’s instructions and syringed through a 1.2 mm blunt needle to disperse cell clumps. Cells were counted using a Vi-Cell XR (Beckman) and 5,000 to 10,000 viable cells (depending on the length of the experiment) were seeded into the wells of a 96-well flat clear bottom black polystyrene TC-treated microplate (Corning). The cells were rested at room temperature for 10 minutes before incubating for 24 hours at 37 °C, 5% CO_2_ prior to treatment.

#### Irradiation

Plated cells were treated with 2 Gy X-ray radiation using an X-RAD 320 (Precision X-Ray). Control was no irradiation. Plates were returned to 37 °C, 5% CO_2_ immediately after treatment.

#### AZD6738 Treatment

Cells were treated with a five-point concentration response (30 μM – 0.3 μM) of AZD6738 in DMSO. Controls were media only, 0.1% DMSO (Sigma) and 1 μl Gemcitabine (Sigma) in sterile water. Drug wash-out was performed by replacing the media in the wells, washing once with 200 μl pre-warmed media. Plates were returned to 37 °C, 5% CO_2_ immediately after washing.

#### Immunostaining

Cells were fixed with 4% paraformaldehyde and stored in Dulbecco’s PBS (Sigma) at 4 °C until required for staining. All plates from a single experiment were stained for *γ*H2AX simultaneously. Cells were permeabilized with 0.1% Triton-X100 (Sigma) and blocked with a solution of 3% BSA (Sigma) in PBS. *γ*H2AX was detected with an overnight 4 °C incubation in a solution (1:2000 in PBS-3% BSA) of an anti-phospho-Histone H2AX (Ser139) antibody (Millipore). Fluorescent endpoints were generated by incubating for 2 hours at room temperature with a solution (1:1000 in PBS-3% BSA) of Alexa Fluor 594 goat anti-mouse IgG (Invitrogen) together with Hoechst 33342 nuclear stain (1:10,000, Invitrogen). Cells were washed and stored in PBS prior to imaging.

#### Caspase-3 Assay

Caspase-3 activity in AZD6738 treated LoVo cells was assessed using the NucView 488 Live Cell Caspase-3 Assay Kit (Biotium), following the manufacturer’s protocol. Cells were treated with 1 μM NucView Caspase-3 substrate immediately before compound dosing and fixed throughout the time course with 4% paraformaldehyde.

#### Imaging

Microplates were imaged at 10x magnification on an ArrayScan VTi (Thermo) using a Compartmental Analysis assay protocol. Exposure, nuclear size range and nuclear segmentation were kept constant for each plate. Nuclei (and cells) were defined as objects with a diameter within a user-defined range and a staining intensity above a user-defined threshold. *γ*H2AX responders were defined for each concentration of AZD6738 as cells with an average nuclear staining level above the user-defined threshold and expressed as a percentage of the total cell count.

### *In vivo* experimental methods

#### *In vivo* studies

Female Swiss nude mice (AP:ONU- Foxn1^nu^; AstraZeneca) were housed in negative pressure isolators (PFI Systems Ltd.) or in an individually ventilated cage system (Tecniplast Ltd.). Experiments were conducted on 8–12 week-old animals in full accordance with the United Kingdom Home Office Animal (Scientific Procedures) Act 1986. Human tumor xenografts were established by subcutaneous injection of 1 × 10^7^ LoVo cells on the flank. Animals were randomized into treatment groups (n = 10–15 per group) when tumors reached a defined palpable size (0.2–0.3 cm^3^). AZD6738, or vehicle, were administered once daily for up to 21 days via oral gavage. IR was administered using a self-contained x-ray system (DMG Control Systems, model Irradiator 320). Tumor targeted IR was administered in fractions of 2 Gy (1.7 minutes) per mouse per day for up to 5 consecutive days. For AZD6738 + IR combination, AZD6738 oral dose was administered 2 hours prior to IR. Tumors were measured up to three times weekly with calipers to calculate mean change in tumor volume.

For pharmacodynamic time course studies, mice were randomized into treatment groups (n = 4–5 per group) when the average tumor volume reached a defined palpable size (0.5–0.7 cm^3^). Mice were dosed with either vehicle or AZD6738 (25 mg/kg single dose, once daily for 4 days or twice daily for 4 days, or 50 mg/kg once daily for 4 days). Sub-groups of animals were humanely killed at multiple time points (2 h, 8 h & 24 h) after final doses. For AZD6738 + IR combination pharmacodynamic studies, mice were administered single doses of either vehicle, AZD6738 (50 mg/kg), IR (2 Gy), or a combination of AZD6738 (50 mg/kg) + IR (2 Gy). Sub-groups of animals were humanely killed at multiple time-points (2 h, 6 h, 24 h & 48 h) after IR dosing.

All animal studies were conducted following an ethical committee review (by the AstraZeneca Animal Welfare and Ethical Review Body or AWERB) and statistical design review in full accordance with the UK Home Office Animal (Scientific Procedures) Act 1986 and EU Directive 2010/60 and AstraZeneca Global Bioethics policy. All experimental protocols where approved by the UK secretary of state for the home office and permission to carry out this work was granted under project license PPL 40/3483.

#### Immunohistochemistry

Tumors were fixed in 10% neutral buffered formalin (NBF) for 24–48 hrs, then processed and embedded in paraffin wax. 4 *μ*m sections were cut onto electrostatically charged glass slides (Colorfrost®), de-waxed in xylene, and re-hydrated through graded alcohols. Heat induced antigen retrieval was performed using Dako Target Retrieval Solution (pH9.0) in a RHS-2 microwave (Milestone, Italy) at 110 °C for 2 minutes. Endogenous peroxide activity was blocked by immersion in 3% hydogen peroxide for 10 minutes and sections were subsequently incubated for 20 minutes with Background Blocker with Casein (A.Menarini Diagnostics Ltd, Berkshire) to reduce non-specific background staining. Immunohistochemistry was performed using a Labvision auto-stainer with X-Cell Plus polymer detection system (A.Menarini Diagnostics Ltd, Berkshire). Slides were incubated with rabbit polyclonal anti-phospho-histone H2AX (Ser139) Antibody (Cell signaling Technology), diluted 1:300 for 60 minutes at room temperature. Immunoreactivity was visualized using 3,3′ Diaminobenzidine (DAB) and slides subsequently counter-stained with haematoxylin.

#### Imaging

Slides were digitized using the Scanscope XT slide scanner (Aperio, Vista, CA, USA) at 20x magnification and quantified using the Spectrum Analysis package and Image Scope viewing software (Aperio, Vista, CA, USA). Xenograft images were manually delineated to ensure *γ*H2AX staining only in areas of viable tumor was quantified. A nuclear detection algorithm was used to quantify the numbers of brown, DAB labeled, positive nuclei and the numbers of blue, haematoxylin stained negative nuclei. The number of positive nuclei present in the image were expressed as a percentage of the total number of positive and negative nuclei.

### Mathematical modeling

The *in vitro* model was developed using the commercial software package Matlab (The MathWorks, Inc., Natick, Massachusetts, United States). *In vivo* model development was performed using the commercial ACSLX v2.5.0.6 software package (AEgis Technologies Group, Inc). Source code is included in Supplementary Information.

Under *in vitro* conditions, drug concentration is considered constant and set to zero at the relevant time point to simulate drug wash-out. *In vivo* drug concentration is represented by a standard two-compartment PK model (see supplementary materials, equations 44–47).

*γ*H2AX positive cells in the model was calculated as the number of cells in the S damaged state (see Fig. 1) as a fraction of the total population (G1, S, and G2/M cells), multiplied by a scaling factor *z* to accommodate differences between experimental *in vitro* methods, resulting from manual thresholding by the operator which can be subject to variation between experiments, and the numerical simulations (see supplementary materials, equation 14).

Instantaneous DNA damage by IR was modeled by initiating the cell population in the appropriate IR damaged phase (see Supplementary Table 1 for model initial conditions) and transition into the corresponding undamaged cell cycle phase. The percentage of *γ*H2AX positive cells resulting from IR induced damage was calculated as the total number of cells occupying the IR damaged versions of the cell cycle compartments as a fraction of the total cell population (see supplementary materials, equation 43).

#### Parameter Estimation

Parameter estimation was performed using the control vector parameterization (direct and sequential) method^49^,^50^, in the J2 software package (RES Group Inc, Boston, MA). The weighted least squares objective function was employed using equal weighting for all data. The underlying non-linear programming problem in the parameter estimation was solved using successive quadratic programming and the necessary partial derivatives of the objective function with respect to the estimated parameters was computed using the staggered corrector forward sensitivity method^51^. Uniqueness of the estimated parameters was assessed in an identifiability analysis described in the following section (see Supplementary Table 2 for parameter values).

#### Parameter Identifiability

Parameter identifiability was assessed using multi-start parameter estimation in the J2 software package (RES Group, Inc, Boston, MA). Parameter estimations were performed from 3,000 randomly generated starting values for the estimated parameters, with uniform and independent sampling (in log space) between wide upper and lower bounds (see Supplementary Information section 4, and Supplementary Figures 1 and 2).

### Model Statistics

Goodness of fit was assessed using R^2^. OriginPro (Origin (OriginLab, Northampton, MA)) was used to perform a linear fit with the slope fixed to a value of 1. See Supplementary Table 3 and Supplementary Figures 7–10 for R, R^2^ values, and parity plots for the *in vitro* experiments in Figs 2 and 3.

### *In vitro* Image Analysis

*In vitro* image analysis was performed using the HCS Analyzer software package, version 1.2.5^52^.

## Additional Information

**How to cite this article**: Checkley, S. *et al.* Bridging the gap between *in vitro* and *in vivo*: Dose and schedule predictions for the ATR inhibitor AZD6738. *Sci. Rep.* **5**, 13545; doi: 10.1038/srep13545 (2015).

## Supplementary Material

Supplementary Information

Supplementary Information
