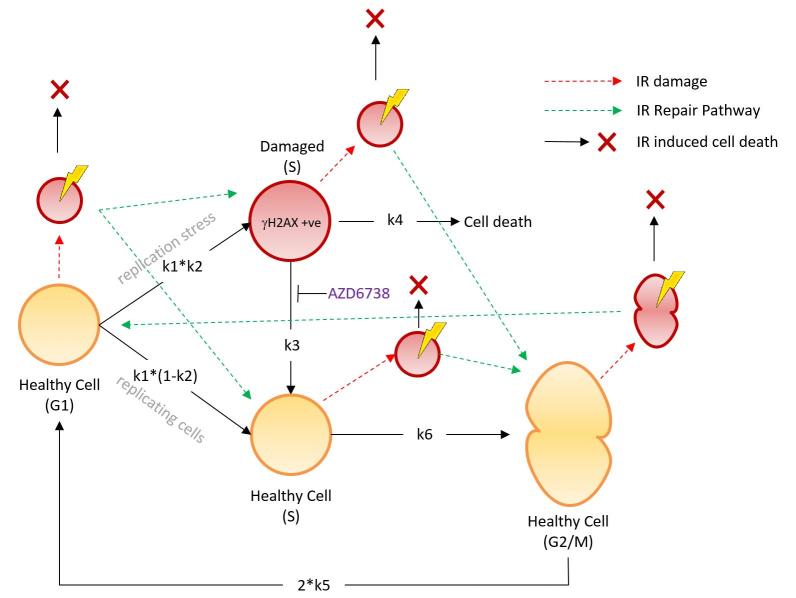# Corrigendum: Bridging the gap between *in vitro* and *in vivo*: Dose and schedule predictions for the ATR inhibitor AZD6738

**DOI:** 10.1038/srep16545

**Published:** 2016-02-09

**Authors:** Stephen Checkley, Linda MacCallum, James Yates, Paul Jasper, Haobin Luo, John Tolsma, Claus Bendtsen

Scientific Reports 5: Article number: 1354510.1038/srep13545; published online: 08272015; updated: 02092016

In the Supplementary Information file originally published with this Article, parameter k6 was omitted from Table 2. This error has been corrected in the Supplementary Information that now accompanies the Article.

In addition, the model source codes were omitted.

There was also an error in Fig. 1, where reaction ‘k5’ now reads ‘2*k5’. The correct Fig. 1 appears below.

These errors have now been corrected in both the PDF and HTML versions of the Article.

## Figures and Tables

**Figure 1:**