# Supplementary material for: Bridging the gap between in vitro and in vivo: Dose and schedule predictions for the ATR inhibitor AZD6738
Source: Sci Rep. 2015 Aug 27;5:13545. doi: 10.1038/srep13545 (PMC4550834; doi:10.1038/srep13545)
Supplement: Supplementary Information [file srep13545-s1.pdf]

Bridging the gap between *in vitro* and *in vivo*: Dose and schedule predictions for the ATR inhibitor AZD6738. Supplementary Information.

Stephen Checkley[1,*], Linda MacCallum[1], James Yates[1], Paul Jasper[2], Haobin Luo[2], John Tolsma[2], and Claus Bendtsen[1,*]

[1]AstraZeneca, Alderley Park, Macclesfield, SK10 4TG. UK

[2]RES Group Inc. Boston, MA. USA

[*]scheckley@gmail.com, claus.bendtsen@astrazeneca.com

23rd June 2015

1 Model Equations

The set of ordinary differential equations used to describe the temporal evolution of the cell population are detailed below:

$$\frac{dG1}{dt} = 2 \cdot k5 \cdot G2 - k1 \cdot k2 \cdot G1 - k1(1 - k2) \cdot \mathrm{G1} + 2 \cdot \mathrm{k_{IRrepair}} \cdot \mathrm{G2_{IR}} \tag{1}$$

$$\frac{dS}{dt} = k1 \cdot (1 - k2) \cdot G1 + (k3 \cdot Sd) \cdot \left(1 - \frac{1}{1 + (\frac{\mathrm{ATR}}{\mathrm{ki}})^h}\right) - k5 \cdot S + (1 - k2) \cdot \mathrm{k_{IRrepair}} \cdot \mathrm{G1_{IR}} \tag{2}$$

As replication stress is believed to be primarily encountered during DNA synthesis in S phase, cells transitioning from G1 to S are subject to a rate of damage (k2), transitioning from G1 to an S "damaged" (Sd) phase, parallel to the healthy S phase in the cell cycle.

$$\frac{dSd}{dt} = k1 \cdot k2 \cdot G1 - k4 \cdot Sd - (k3 \cdot Sd) \cdot \left(1 - \frac{1}{1 + (\frac{\mathrm{ATR}}{\mathrm{ki}})^h}\right) + k2 \cdot \mathrm{k_{IRrepair}} \tag{3}$$

ATR inhibition prevents cells initiating DNA repair which, in this model, accumulates during S phase. Therefore the transition from the S damaged phase (k3) is inhibited when compound is added to the model. Cells delayed in the damaged state by inhibition of the repair reaction are removed from the

system (at rate k4), simulating apoptosis in response to prolonged damage, and a decrease in the total cell population.

$$\frac{dG2}{dt} = k6 \cdot S - k5 \cdot G2 + \mathrm{k_{IRrepair}} \cdot \mathrm{S_{IR}} + \mathrm{k_{IRrepair}} \cdot \mathrm{Sd_{IR}} \tag{4}$$

$$\frac{\mathrm{dApoptosis}}{dt} = k4 \cdot \mathrm{Sd} \tag{5}$$

$$\frac{\mathrm{dApoptosis_{IR}}}{dt} = k_{\mathrm{IRapop}} \cdot \mathrm{G1_{IR}} + k_{\mathrm{IRapop}} \cdot \mathrm{S_{IR}} + k_{\mathrm{IRapop}} \cdot \mathrm{G2_{IR}} + k_{\mathrm{IRapop}} \cdot \mathrm{Sd_{IR}} \tag{6}$$

It was observed *in vitro* that DNA damage from IR occurs instantaneously and therefore a rate of DNA damage resulting from exposure to radiation was not incorporate into the model. Instead, the initial conditions were set such that the population of cells begins in the damaged version of the cell cycle stages and is repaired at the beginning of the time course simulation. This approach enabled the separation of damage resulting from replication stress from damage induced by radiation, and also separate the repair mechanisms so that ATR inhibition was specific to repairing replication stress induced damage.

$$\frac{dG1_{\mathrm{IR}}}{dt} = -k_{\mathrm{IRrepair}} \cdot \mathrm{G1_{IR}} - k_{\mathrm{IRapop}} \cdot G1_{\mathrm{IR}} \tag{7}$$

$$\frac{dSd_{\mathrm{IR}}}{dt} = -k_{\mathrm{IRrepair}} \cdot \mathrm{Sd_{IR}} - k_{\mathrm{IRapop}} \cdot \mathrm{Sd_{IR}} \tag{8}$$

$$\frac{dS_{\mathrm{IR}}}{dt} = -k_{\mathrm{IRrepair}} \cdot \mathrm{S_{IR}} - k_{\mathrm{IRapop}} \cdot \mathrm{S_{IR}} \tag{9}$$

$$\frac{dG2_{\mathrm{IR}}}{dt} = -k_{\mathrm{IRrepair}} \cdot \mathrm{G2_{IR}} - k_{\mathrm{IRapop}} \cdot \mathrm{G2_{IR}} \tag{10}$$

$$\frac{\mathrm{dATRi}}{dt} = \mathrm{koff} \cdot \mathrm{ATR\_\ ATRi} - \mathrm{kon} \cdot \mathrm{ATR} \cdot \mathrm{ATRi} \tag{11}$$

$$\frac{\mathrm{dATR}}{dt} = \mathrm{ATRi\_ATRi} \cdot \mathrm{koff} - \mathrm{kon} \cdot \mathrm{ATR} \cdot \mathrm{ATRi} \tag{12}$$

$$\text{total cells} = G1 + G2 + S + Sd \tag{13}$$

$$\gamma\mathrm{H2AX} = \frac{Sd}{\text{total cells}} \cdot 100 \cdot z \tag{14}$$

Drug was represented with the variable ATRi in the model, and drug binding was modeled using mass action kinetics. Drug concentration was considered to be constant throughout the *in vitro* experiments. Drug binding to target *in vitro* was represented with the reversible reaction (equations 11 and 12).

During the development of the model replication stress and IR damage repair was modeled by damaged cells transitioning back to the same cell cycle phase or into to the next phase. Parameter fitting of both

of these approaches returned the same parameter sets and both models were capable of simulating the experimental data. It was therefore concluded that the route taken by repaired cells transitioning back into the cell cycle does not make a significant difference to the behavior of the model.

1.1 Integration of the cell cycle model with a structural model of tumor growth

Xenografted tumor volume and γH2AX pharmacodynamics were predicted by implementing the *in vitro* based cell cycle model into a semi-mechanistic model of tumor pathophysiology [1]. The tumor model assumes an avascular tumor mass with oxygen, drug and nutrients being delivered by blood vessels in the surrounding host tissue, a common phenotype for xenografted tumors. [2]. The model comprises two physical compartments: a proliferating outer shell (super-script S) and a quiescent inner core (super-script C). The size of the proliferating shell is set by the current tumor volume and the thickness of the shell (R_{diff}). As the tumor volume evolves over time, mass transfers between the shell and the core to maintain a constant shell thickness. A copy of the cell cycle model was implemented in both of the physical compartments with the fluxes associated with cell cycle progression being set to zero in the core. Mass transfer between the two physical compartments was implemented with mass moving from each state of the cell cycle proportional to the volume in the donor compartment this state occupied.

The equations for the tumor shell are as follows:

$$\frac{\text{dG1}^{\text{S}}}{dt} = 2 \cdot k5 \cdot G2^S - k1 \cdot k2 \cdot G1^S - k1(1-k2) \cdot G1^S + 2 \cdot k_{IRrepair} \cdot G2^S_{IR} - k_{\text{Transfer}} \cdot f_{G1} \quad (15)$$

$$\frac{dS^S}{dt} = k1 \cdot (1-k2) \cdot G1^S + (k3 \cdot Sd^S) \cdot \left(1 - \frac{1}{1 + (\frac{\text{ATR}}{\text{ki}})^h}\right) - k6 \cdot S^S + (1-k2) \cdot k_{\text{IRrepair}} \cdot G1^S_{IR} - k_{\text{Transfer}} \cdot f_S \quad (16)$$

$$\frac{dSd^S}{dt} = k1 \cdot k2 \cdot G1^S - k4 \cdot Sd^S - (k3 \cdot Sd^S) \cdot \left(1 - \frac{1}{1 + (\frac{\text{ATR}}{\text{ki}})^h}\right) + k2 \cdot k_{\text{IRrepair}} - k_{\text{Transfer}} \cdot f_{Sd} \quad (17)$$

$$\frac{dG2_S}{dt} = k6 \cdot S^S - k5 \cdot G2^S + k_{\text{IRrepair}} \cdot S^S_{\text{IR}} + k_{\text{IRrepair}} \cdot Sd^S_{\text{IR}} - k_{\text{Transfer}} \cdot f_{G2} \quad (18)$$

where f_i is defined below for each state.

The delay between cells (either background replication stress or IR induced) entering apoptosis and finally being removed from the tumor volume is modeled using a series of transit compartments where A1, A2 and A3 are states for dying cells:

$$\frac{dA1^S}{dt} = k4 \cdot \mathrm{Sd} + k_{\mathrm{IRapop}} \cdot \mathrm{G1}_{\mathrm{IR}} + k_{\mathrm{IRapop}} \cdot \mathrm{S}_{\mathrm{IR}} + k_{\mathrm{IRapop}} \cdot G2_{\mathrm{IR}} + k_{\mathrm{IRapop}} \cdot \mathrm{Sd}_{\mathrm{IR}} - \mathrm{Kex} \cdot A1^S - k_{\mathrm{Transfer}} \cdot f_{A1} \quad (19)$$

$$\frac{dA2^S}{dt} = \mathrm{Kex} \cdot (A1^S - A2^S) - k_{\mathrm{Transfer}} \cdot f_{A2} \quad (20)$$

$$\frac{dA3^S}{dt} = \mathrm{Kex} \cdot (A2^S - A3^S) - k_{\mathrm{Transfer}} \cdot f_{A3} \quad (21)$$

The following equations represent the IR damaged compartments:

$$\frac{dG1^S_{\mathrm{IR}}}{dt} = -k_{\mathrm{IRrepair}} \cdot G1^S_{\mathrm{IR}} - k_{\mathrm{IRapop}} \cdot G1^S_{\mathrm{IR}} - k_{\mathrm{Transfer}} \cdot f_{G1} \quad (22)$$

$$\frac{dSd^S_{\mathrm{IR}}}{dt} = -k_{\mathrm{IRrepair}} \cdot Sd^S_{\mathrm{IR}} - k_{\mathrm{IRapop}} \cdot Sd^S_{\mathrm{IR}} - k_{\mathrm{Transfer}} \cdot f_{Sd} \quad (23)$$

$$\frac{dS^S_{\mathrm{IR}}}{dt} = -k_{\mathrm{IRrepair}} \cdot S^S_{\mathrm{IR}} - k_{\mathrm{IRapop}} \cdot S^S_{\mathrm{IR}} - k_{\mathrm{Transfer}} \cdot f_{S} \quad (24)$$

$$\frac{dG2^S_{\mathrm{IR}}}{dt} = -k_{\mathrm{IRrepair}} \cdot G2^S_{\mathrm{IR}} - k_{\mathrm{IRapop}} \cdot G2^S_{\mathrm{IR}} - k_{\mathrm{Transfer}} \cdot f_{G2} \quad (25)$$

For the necrotic (non-replication) core of the tumor (designated with super-script "C"), the equations are the same as above but for the removal of cell cycle progression. DNA repair is assumed to still occur with the consequence that IR repairing G2/M cells transit back to G1.

$$\frac{dG1^C}{dt} = 2 \cdot k_{\mathrm{IRrepair}} \cdot G2^C_{\mathrm{IR}} + k_{\mathrm{Transfer}} \cdot f_{G1} \quad (26)$$

$$\frac{dS^C}{dt} = (k3 \cdot Sd^C) \cdot \left(1 - \frac{1}{1 + (\frac{\text{ATR}}{\text{ki}})^h}\right) + (1 - k2) \cdot k_{\text{IRrepair}} \cdot G1^{\text{IR}} + k_{\text{Transfer}} \cdot f_S \tag{27}$$

$$\frac{dSd^C}{dt} = -(k3 \cdot Sd^C) \cdot \left(1 - \frac{1}{1 + (\frac{\text{ATR}}{\text{ki}})^h}\right) + k2 \cdot k_{\text{IRrepair}} + k_{\text{Transfer}} \cdot f_{Sd} \tag{28}$$

$$\frac{dG2^C}{dt} = +k_{\text{IRrepair}} \cdot S^C_{\text{IR}} + k_{\text{IRrepair}} \cdot Sd^C_{\text{IR}} + k_{\text{Transfer}} \cdot f_{G2} \tag{29}$$

The delay between cells (either background replication stress or IR damage induced) that are entering apoptosis and finally being removed from the tumor volume is modeled using a series of transit compartments:

$$\frac{dA1^C}{dt} = k4 \cdot Sd^C + k_{\text{IRapop}} \cdot G1^C_{\text{IR}} + k_{\text{IRapop}} \cdot S^C_{\text{IR}} + k_{\text{IRapop}} \cdot G2^C_{\text{IR}} + k_{\text{IRapop}} \cdot Sd^C_{\text{IR}} - \text{Kex} \cdot A1^C + k_{\text{Transfer}} \cdot f_{A1} \tag{30}$$

$$\frac{dA2^C}{dt} = \text{Kex} \cdot (A1^C - A2^C) + k_{\text{Transfer}} \cdot f_{A2} \tag{31}$$

$$\frac{dA3^C}{dt} = \text{Kex} \cdot (A2^C - A3^C) + k_{\text{Transfer}} \cdot f_{A3} \tag{32}$$

The following equations represent the IR damaged compartments in the tumor core:

$$\frac{dG1^C_{\text{IR}}}{dt} = -k_{\text{IRrepair}} \cdot G1^C_{\text{IR}} - k_{\text{IRapop}} \cdot G1^C_{\text{IR}} + k_{\text{Transfer}} \cdot f_{G1} \tag{33}$$

$$\frac{dSd^C_{\text{IR}}}{dt} = k_{\text{IRrepair}} \cdot Sd^C_{\text{IR}} - k_{\text{IRapop}} \cdot Sd^C_{\text{IR}} + k_{\text{Transfer}} \cdot f_{Sd} \tag{34}$$

$$\frac{dS^C_{\textbf{IR}}}{dt} = -k_{\text{IRrepair}} \cdot S^C_{\text{IR}} - k_{\text{IRapop}} \cdot S^C_{\text{IR}} + k_{\text{Transfer}} \cdot f_S \tag{35}$$

$$\frac{dG2^C_{\text{IR}}}{dt} = -k_{\text{IRrepair}} \cdot G2^C_{\text{IR}} - k_{\text{IRapop}} \cdot G2^C_{\text{IR}} + k_{\text{Transfer}} \cdot f_{G2} \tag{36}$$

ATR inhibition is assumed constant throughout the tumor volume.

The shell volume is calculated as:

$$V^{\text{shell}} = G1^S + Sd^S + G2^S + S^S + A1^S + A2^S + A3^S + G1^S\text{IR} + G2^S_{\text{IR}} + Sd^S_{\text{IR}} + S^S_{\text{IR}} \quad (37)$$

The core volume is calculated as:

$$V^{\text{core}} = G1^C + Sd^C + G2^C + S^C + A1^C + A2^C + A3^C + G1^C_{\text{IR}} + G2^C_{\text{IR}} + Sd^C_{\text{IR}} + S^C_{\text{IR}} \quad (38)$$

and so the total volume that is compared to the observed tumor volume data is calculated using the following equation:

$$V = V^{shell} + V^{core} \quad (39)$$

When the total volume of the tumor is such that its radius (R), calculated using:

$$R_{\text{total}} = (\frac{3V}{4\pi})^{\frac{1}{3}} \quad (40)$$

is larger than the diffusion thickness R_{diff} then the total rate of mass transfer between the shell and the core (k_{Transfer}) is calculated as:

$$k_{\text{transfer}} = \frac{dV}{dt} \cdot \frac{((R_{\text{total}} - R_{\text{diff}})^2}{(R_{\text{total}})} - \text{Kex} \cdot \text{A3}^{\text{C}} \quad (41)$$

where for following equation:

$$\frac{dV}{dt} = k5 \cdot G2^S + \cdot k_{\text{IRrepair}} \cdot G2^C_{\text{IR}} \cdot k_{\text{IRrepair}} \cdot G2^S_{\text{IR}} - \text{Kex} \cdot (A3^S + A3^C) \quad (42)$$

is the rate of change of the total volume. Transfer between corresponding states of the cell model in the core and the shell are set as the product of the total mass transfer and the fraction of the volume of the donor compartment, f_{i}, the cell cycle state occupies. If k_{transfer} is positive then the shell is the donor compartment (the tumor is growing) otherwise the donor compartment is the core(the tumor is shrinking). The observed *in vivo* γH2AX signal is predicted as:

$$\gamma\text{H2AX} = \frac{(Sd^S + Sd^S_{\text{IR}} + G1^S_{\text{IR}} + S^S_{\text{IR}} + G2^S_{\text{IR}} + Sd^C + Sd^C_{\text{IR}} + G1^C_{\text{IR}} + S^C_{\text{IR}} + G2^C_{\text{IR}})}{V} \cdot z \cdot 100 \quad (43)$$

All states in the cell cycle model are initially set to those of the in the *in vitro* model.

To account for experimental differences between the *in vitro* and *in vivo* quantification of γH2AX positive cells, z scaling factors are applied in the model depending on whether it is simulating *in vitro* or *in vivo* data sets (see table 2).

To simulate *in vivo* drug pharmaco-kinetics and pharmaco-dynamics a standard two-compartment model was used to describe gut, central and peripheral clearance [3].

$$\frac{d\text{GUT}}{dt} = -ka \cdot \text{GUT} \quad (44)$$

$$\frac{d\text{CEN}}{dt} = ka \cdot \text{GUT} - (q + cl) \cdot \frac{\text{CEN}}{v1} + q \cdot \frac{d\text{PER}}{v2} \quad (45)$$

$$\frac{d\text{PER}}{dt} = q \cdot (\frac{\text{CEN}}{v1} - \frac{\text{PER}}{v2}) \quad (46)$$

$$\frac{\text{Cp}}{dt} = \frac{\text{CEN}}{v1} \quad (47)$$

Drug (ATRi) binding was modeled using mass action kinetics where ATR_ATRi represents drug bound to ATR. For *in vivo* scenario Cp represents drug concentration and the following equations are used:

$$\frac{d\text{ATR}}{dt} = \mathit{koff} \cdot \text{ATR\_ATRi} \quad (48)$$

$$\frac{d\text{ATR\_ATRi}}{dt} = \mathit{kon} \cdot \text{ATR} \cdot \mathit{kscale} \cdot \text{Cp} \cdot \mathit{Fu} \quad (49)$$

The scaling factor *kscale* is used to correct the drug concentration for the free fraction after binding to serum albumin and *Fu* to correct for diffusion of drug from the surrounding plasma into the tumor mass.

For the *in vitro* scenario drug concentration is considered to be constant and the following equations used:

$$\frac{d\text{ATR}}{dt} = \mathit{koff} \cdot \text{ATR\_ATRi} \quad (50)$$

$$\frac{d\text{ATR\_ATRi}}{dt} = \mathit{kon} \cdot \text{ATR} \cdot \text{ATRi} \cdot \mathit{kscale} \quad (51)$$

2 Model initial conditions

initial condition	value	source
fraction of cells in G1	0.47	Shi M. *et al* (2008)
fraction of cells in Sd	0.0184	Shi M. *et al* (2008)
total fraction of cell in S (S + Sd)	0.32	Shi M. *et al* (2008)
fraction of cells in G2/M	0.21	Shi M. *et al* (2008)
fraction of cells in S	total S - Sd	
G1	fraction of cells in G1 $\cdot$ cell seed density	
G2	fraction of cells in G2 $\cdot$ cell seed density	
S	fraction of cells in S $\cdot$ cell seed density	
Sd	fraction of cells in Sd $\cdot$ cell seed density	
$G1_{IR}$	fraction of cells in G1 $\cdot$ cell seed density	
$G2_{IR}$	fraction of cells in G2 $\cdot$ cell seed density	
S_{IR}	fraction of cells in S $\cdot$ cell seed density	
Sd_{IR}	fraction of cells in Sd $\cdot$ cell seed density	
G1_IR	0.0	
S_IR	0.0	
Sd_IR	0.0	
apoptosis	0.0	
apoptosis$_{IR}$	0.0	
ATR	0.01 μmol/l	
ATRi	initial dose	
ATR _ATRi	0.0	

Table 1: Model initial conditions for monotherapy and in combination with ionizing radiation.

3 Model parameters

parameter	value	units	data source for fitting
h	2.60217	1/h	in vitro
k1	0.0811185	1/h	in vitro
k2	0.757926	1/h	in vitro
k3	1766.78	1/h	in vitro
k4	0.130153	1/h	in vitro
k5	0.110204	1/h	in vitro
ki	0.153196	1/h	in vitro
z (*in vitro*)	2.95487		in vitro
z (*in vivo*)	0.906667		in vivo
kon	0.117798	1/h	in vitro
koff	0.0290027	1/h	in vitro
Fu	0.288711	1/h	
kscale	0.32909		in vitro
$k_{IRrepair}$	0.018	1/h	IR in vitro
k_{IRapop}	0.039	1/h	IR in vitro
v1	0.101	litres/kg	
v2	9.17	litres/kg	
q	0.880	litres/h/kg	
cl	0.603	litres/h/kg	
ka	0.592	1/hr	

Table 2: Model parameter values obtained by optimization. In vitro refers to the in vitro time course data with and without washout. IR in vitro is the in vitro time course data measured following IR treatment only. PK parameters were determined from mouse PK studies prior to this work.

4 Parameter Identifiability

Identifiability of the model was assessed using multi-start parameter estimation in the J2 software package. As a first step, a parameter sensitivity analysis was performed. J2 computes the parametric sensitivity profiles using the staggered corrector forward sensitivity method [5]. The sensitivities are converted to a scalar metric by evaluating the integral of the absolute value of the profile and are shown in Figure 1

(sorted in decreasing sensitivity of cell count). Sensitivities of both cell count and γH2AX with respect to the estimated parameters are computed.

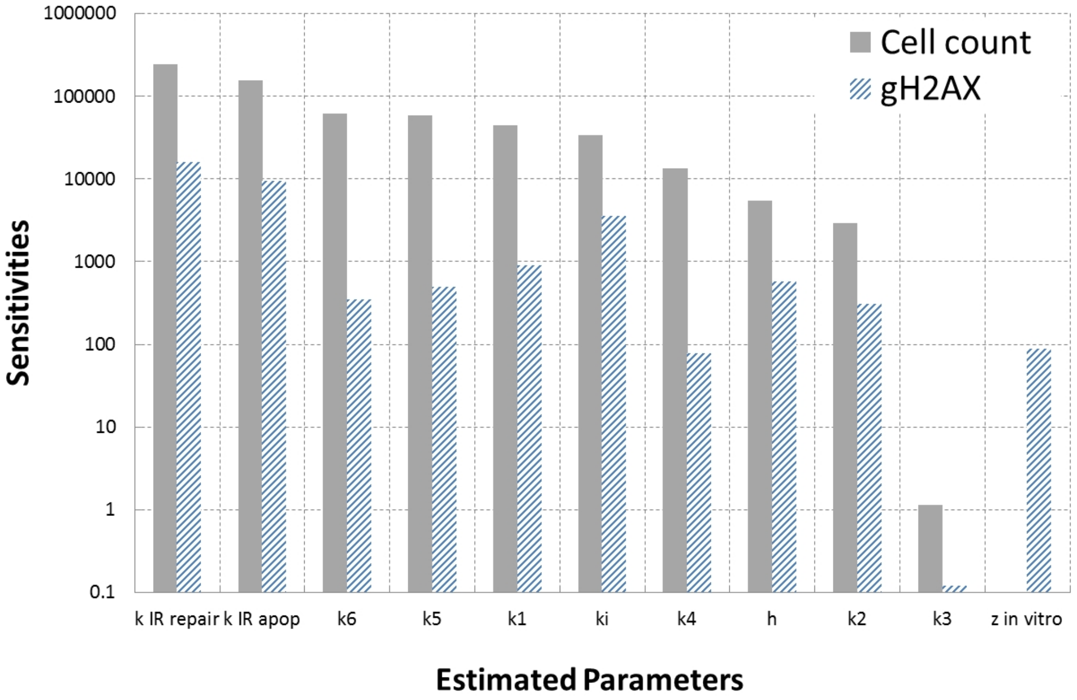

Figure 1: Sensitivity of cell count and γH2AX with respect to estimated parameters.

The sensitivity analysis indicates that all parameters are sensitive and can be estimated from the available in vitro cell count and γH2AX data. To assess identifiability, parameter estimations, fitting in vitro γH2AX and cell count data, were performed from 3,000 randomly generated starting values for the estimated parameters, with uniform sampling (in log space) between upper and lower bounds. The 3,000 sets of estimated parameters were sorted by objective function and the values for the top 50 lowest objective function are shown in Figure 2 (the upper and lower bounds for each parameter can also be seen on the y-axis). The parameter plots are sorted from left to right and top to bottom by decreasing value of sensitivity of cell count. What can be observed in this Figure is that almost all parameters converge to the same optimal fit. Parameters k_i and k_3 were found to be highly correlated, however, the model predictions were found to be insensitive to the different solutions obtained for these two parameters. Given the uniqueness of the estimated parameters, aside from the two highly correlate parameters, we conclude that the best fit parameters were obtained and that the model is identifiable and well constrained by the calibration data.

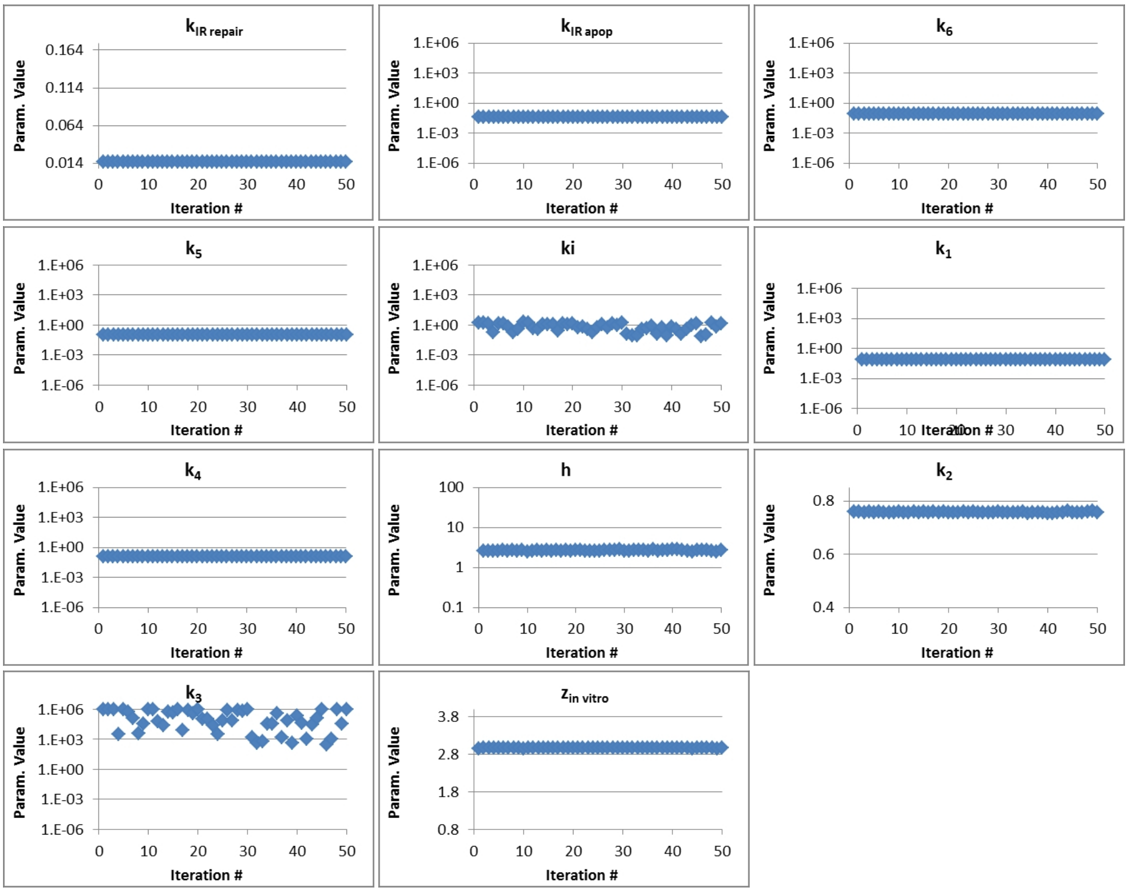

Figure 2: Parameter identifiability summary.

5 Cell Cycle Distribution

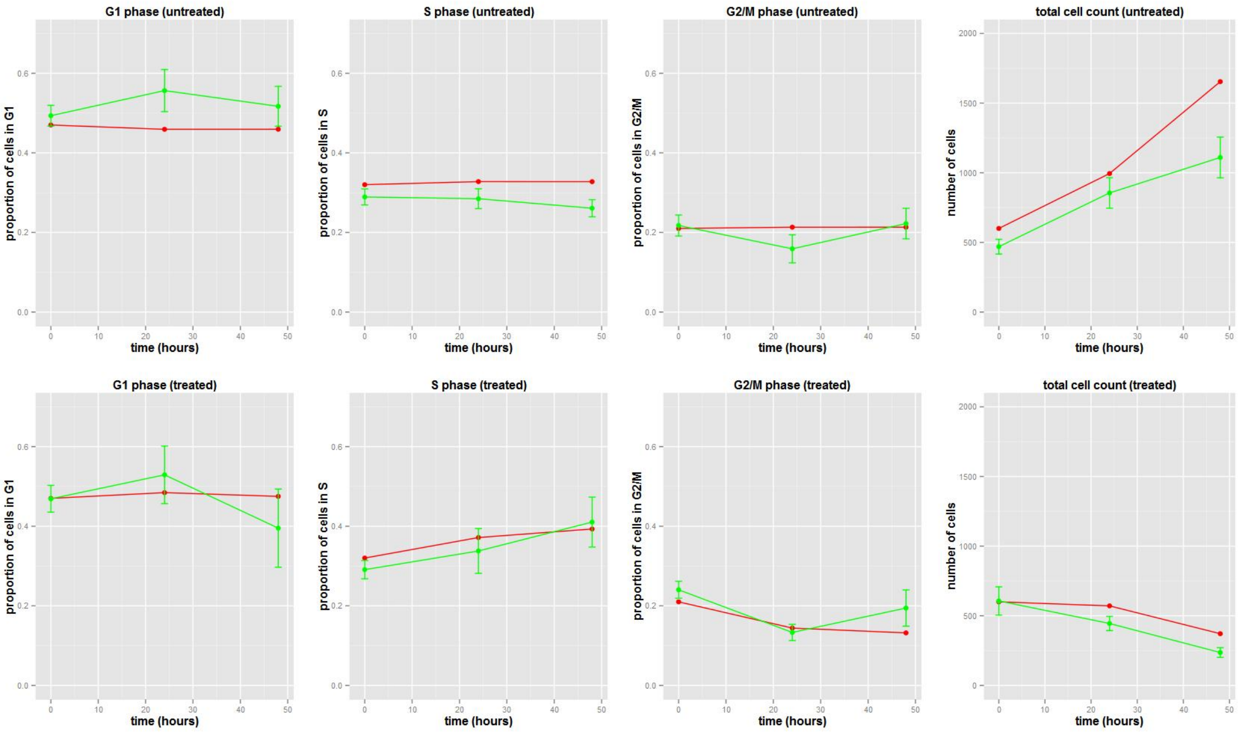

Figure 3: Time course evolution of cell cycle phase distribution, obtained from image analysis performed on in vitro data. Red lines represent model simulations with 10µM AZD6738 and green lines represent experimental observations with 10µM AZD6738. Error bars represent standard deviation of the mean with n=16.

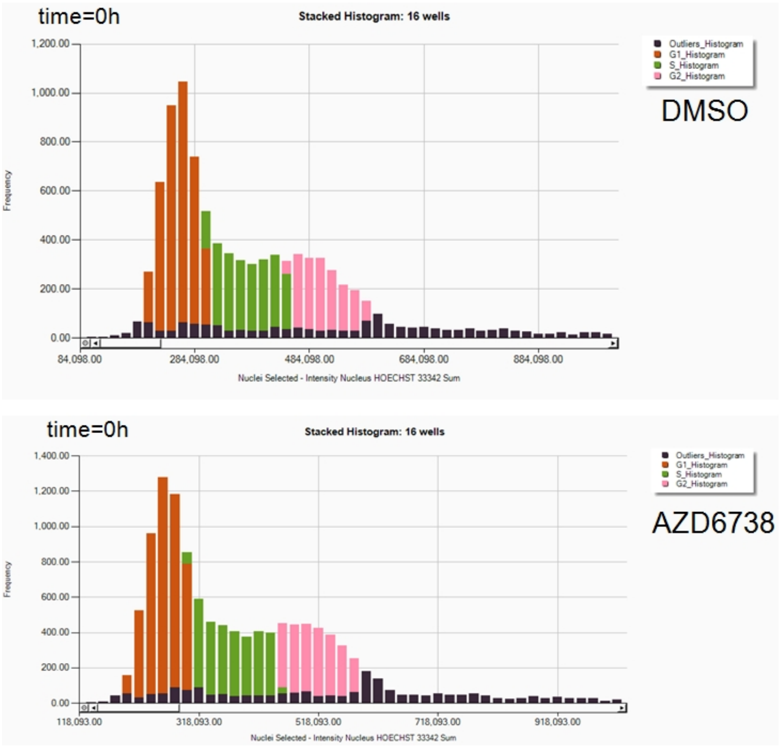

Figure 4: Stacked histogram of G1, S, and G2 cell cycle phase distribution identified using HPC analyzer software. Analysis of time 0 hours. Top figure represents DMSO vehicle control, bottom figure represents AZD6738 treated cells.

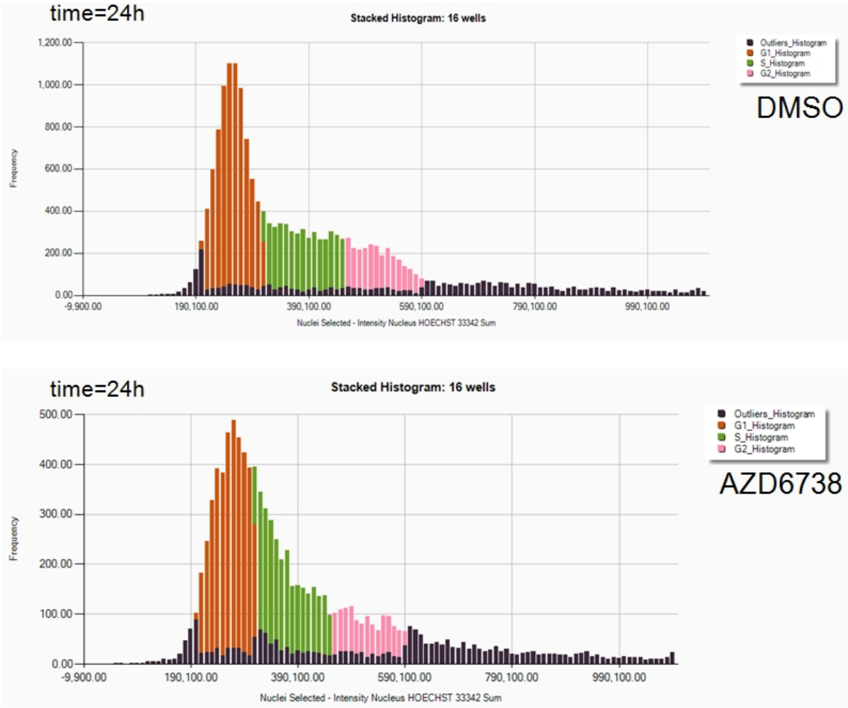

Figure 5: Stacked histogram of G1, S, and G2 cell cycle phase distribution identified using HPC analyzer software. Analysis of 24 hours post treatment. Top figure represents DMSO vehicle control, bottom figure represents AZD6738 treated cells.

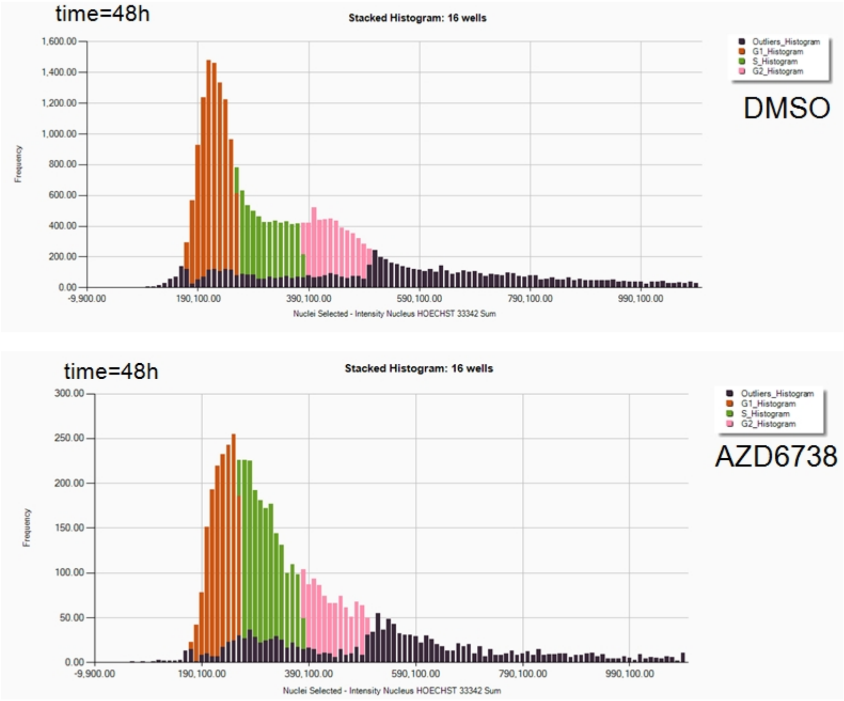

Figure 6: Stacked histogram of G1, S, and G2 cell cycle phase distribution identified using HPC analyzer software. Analysis of 48 hours post treatment. Top figure represents DMSO vehicle control, bottom figure represents AZD6738 treated cells.

6 Model Statistics

R and R^2 values for the *in vitro* model are detailed in table 3.

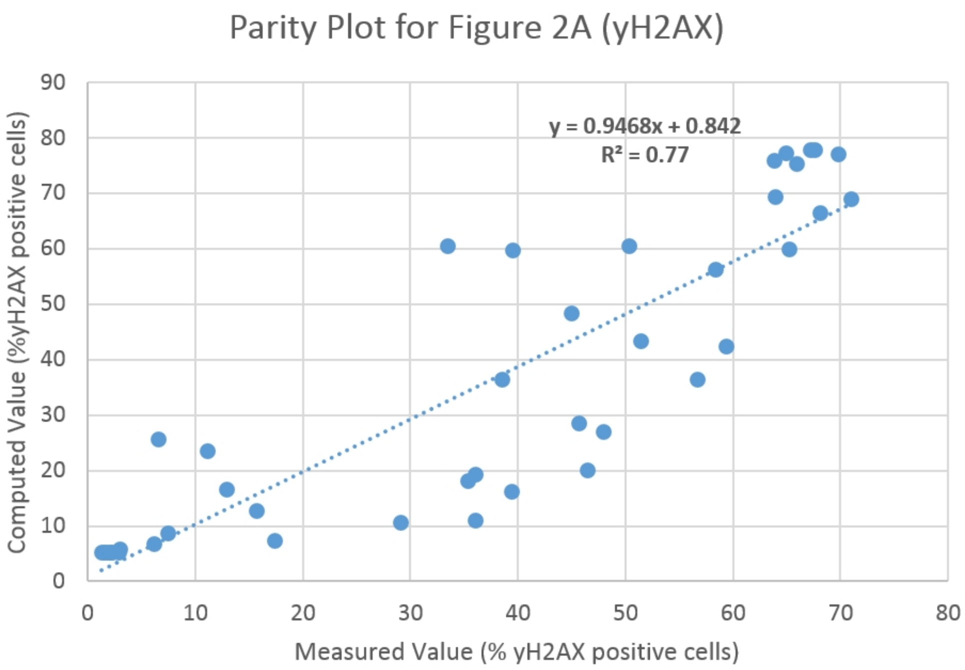

Figure 7: Parity plot of model and experimental data sets shown in Figure 2A.

Figure	R value	R^2 value
2A	0.88	0.77
2B	0.85	0.72
3A	0.93	0.87
3B	0.93	0.87

Table 3: R and R^2 values for figures 2 and 3.

Parity plots for model simulations and experimental data sets are detailed in figures 7-10.

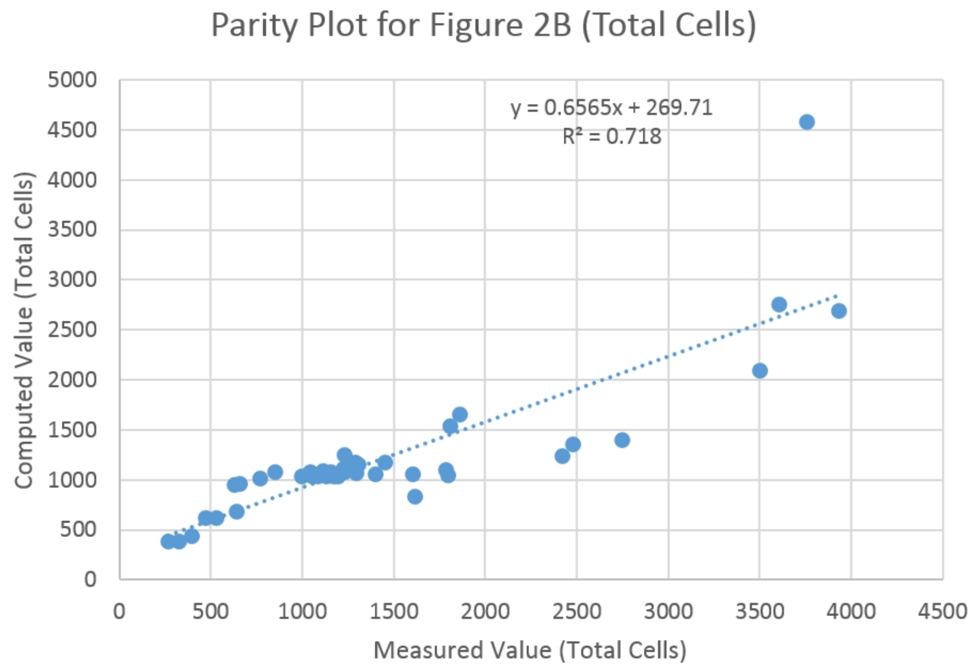

Figure 8: Parity plot of model and experimental data sets shown in Figure 2B.

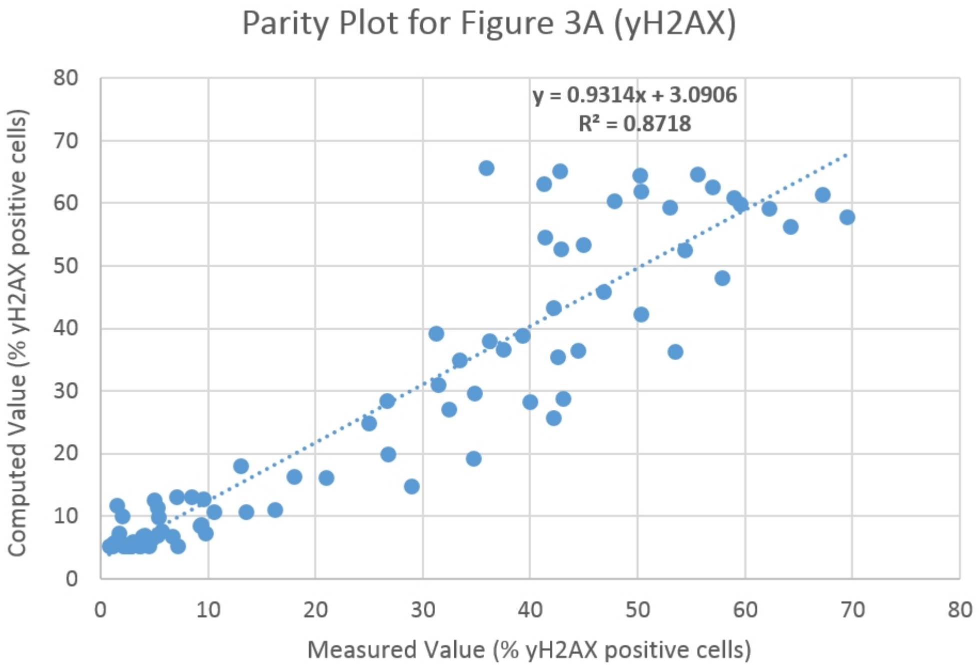

Figure 9: Parity plot of model and experimental data sets shown in Figure 3A.

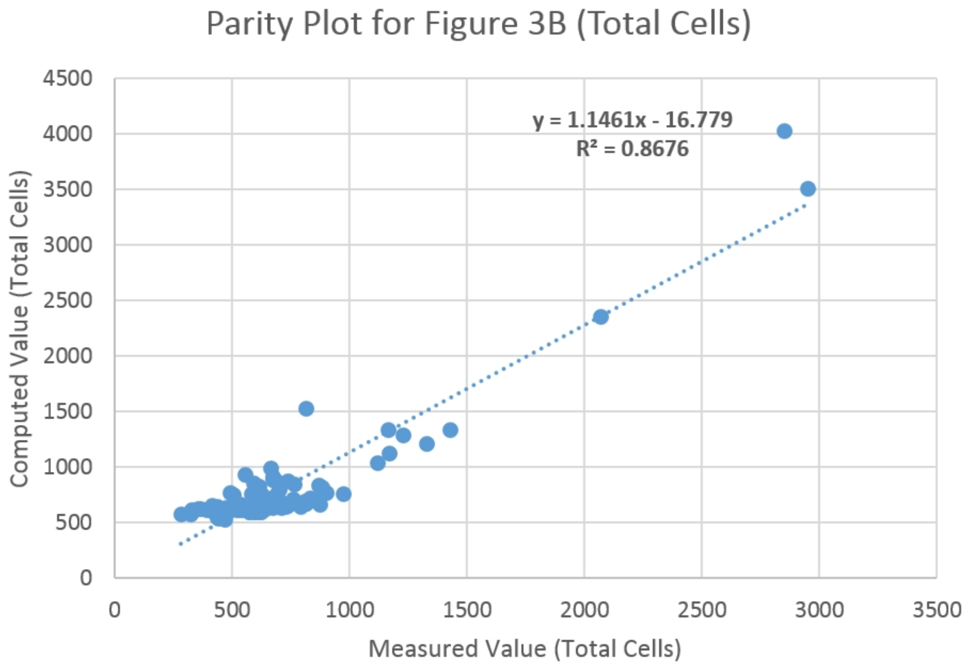

Figure 10: Parity plot of model and experimental data sets shown in Figure 3B.

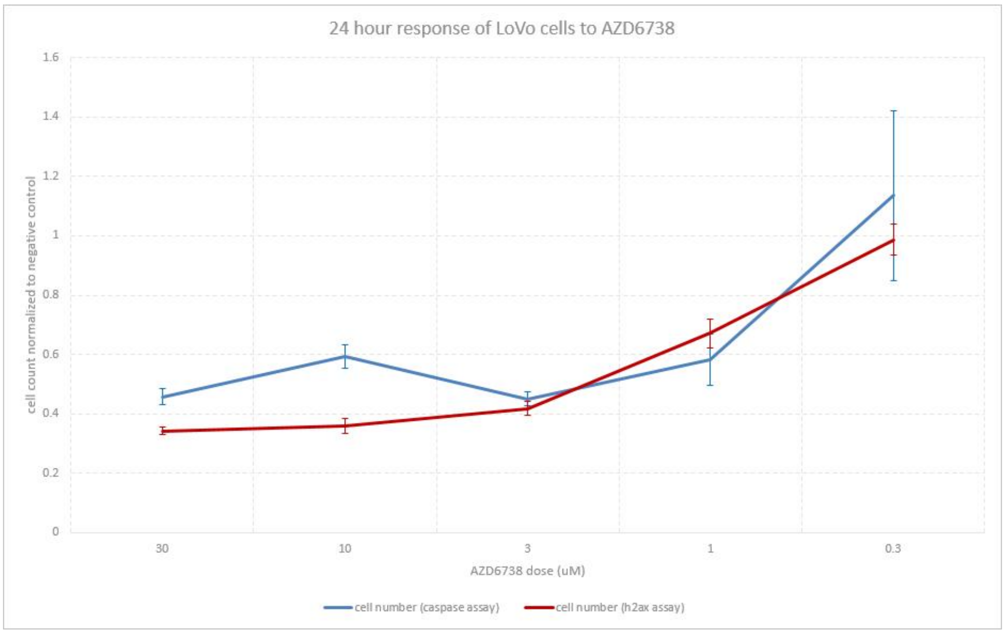

Figure 11: AZD6738 dose response following treatment of LoVo cells for 24 hours. The blue line represents total LoVo cell count recorded during the Caspase assay. Points represent experimental observations at each dose, lines represent standard error (n=4). The red line represents total cell count recorded in the γH2AX assay (n=6) reported in figure 2a and is included for comparison with the data from the Caspase assay. The data is normalized to the negative control (no drug). The caspase assay was performed separately from the γH2AX assay described in the manuscript, however the cell count dose response behavior is consistent between experiments.

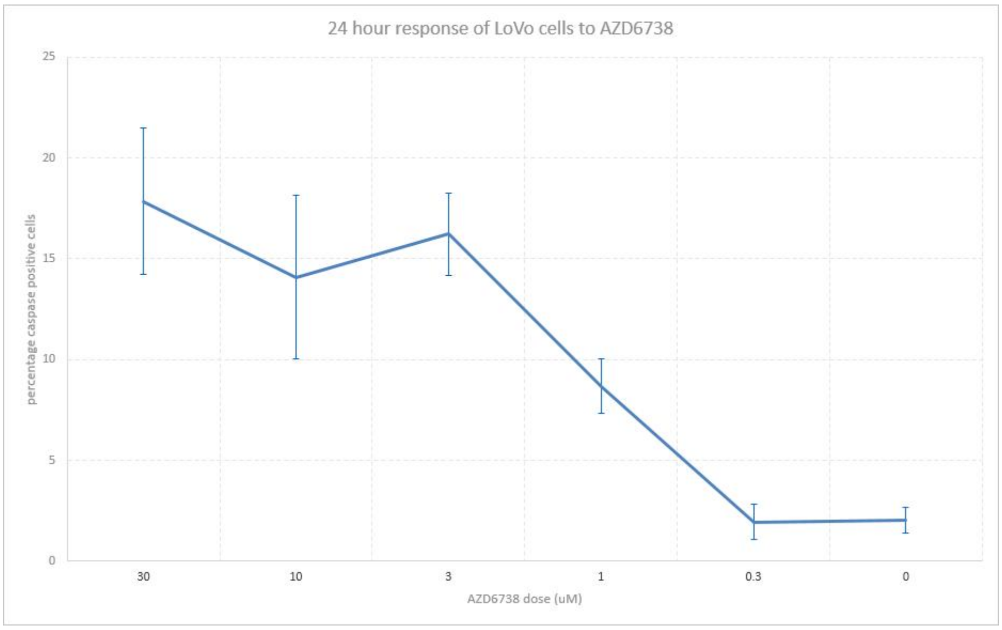

Figure 12: AZD6738 dose response measuring Caspase 3. Points represent experimental observations of LoVo cells following treatment with AZD6738 for 24 hours exposure, lines represent standard error (n=4).

References

[1] Evans, N. D., Dimelow, R. J. & Yates, J. W. Modelling of tumour growth and cytotoxic effect of docetaxel in xenografts. *Comput. Methods Programs Biomed.* **114**, 3–13 (2014).

[2] Smith, N. R. *et al.* Tumour-stromal architecture can define the intrinsic tumour response to VEGF-targeted therapy. *Clin. Cancer Res.* **19**, 6943–56 (2013).

[3] Wagner, J. G. *Pharmacokinetics for the Pharmaceutical Scientist* (CRC Press, 1993).

[4] Shi, M.-D. *et al.* Inhibition of cell-cycle progression in human colorectal carcinoma Lovo cells by andrographolide. *Chem. Biol. Interact.* **174**, 201–10 (2008).

[5] Feehery, W. F., Tolsma, J. E. & Barton, P. I. Efficient sensitivity analysis of large-scale differential-algebraic systems. *Appl. Numer. Math.* **25**, 41–54 (1997).
